# Novel heterotopic continental ileal reservoir based on the Mitrofanoff principle: a case report and review of the literature

**DOI:** 10.3389/fsurg.2023.1202649

**Published:** 2023-09-29

**Authors:** Hou Yakun, Dong Guofan, Liu Qiang, Bahtiyar Azat, Mulati Rexiati, Wang Yujie, Wang Wenguang

**Affiliations:** Medical Center of Urology, The First Affiliated Hospital of Xinjiang Medical University Xinjiang Clinical Medical Research Center of Urogenital Diseases, Urumqi, China

**Keywords:** heterotopic controlled bladder, bladder cancer, short-term efficacy, uroflow diversion, appendix

## Abstract

For patients undergoing radical cystectomy with standard lymphadenectomy for bladder cancer, appropriate urinary diversion (with a pouch and conduit) improves postoperative quality of life, reduces postoperative complications, and prolongs survival. We developed a novel heterotopic ileal reservoir to achieve these goals. This report describes the methodology involved and the incidence of intraoperative and postoperative complications. Three patients who underwent novel heterotopic ileal reservoir creation following radical cystectomy and standard lymphadenectomy (for bladder cancer) were evaluated. The ileum served as a pouch in which the ureters and appendix were implanted by extramural tunnelling. The appendix served as a conduit and pelvic reperitonealization was performed. Operative times, intraoperative blood loss, time to intestinal function recovery, incidence of intestinal obstruction and ureteric reflux, and bladder volumes and continence levels were evaluated. The surgical intervention was successful with operation times ranging 410–525 min, blood loss ranging 300–700 ml, and recovery time for intestinal function ranging 3–5 days. The postoperative hospitalization time was 11–15 days. Subileus occurred in patient B, who recovered after fasting and fluid replacement. Patients B and C achieved complete continence 6 weeks after surgery, while patient A experienced umbilical urine leakage with catheterization time intervals that exceeded 4 h. At 3 months after surgery, the bladder capacities of all patients ranged 250–370 ml. Follow-up cystography suggested the presence of bilateral ureteral reflux in patient A, with mild and moderate reflux on the left right sides, respectively. All patients achieved complete continence. Patients were followed for 3–9 months postoperatively; chest and abdominal computed tomography and cystography showed absence of hydronephrosis, recurrence, or distant metastasis during this period. The novel heterotopic continent ileal reservoir described in this study may be suitable for selected patients. The surgical procedure is safe when performed by well-trained and highly experienced urologists.

## Introduction

Urinary diversion procedures have been performed for more than 200 years and include numerous surgical techniques such as the creation of orthotopic neobladders, the creation of heterotopic continent reservoirs, and non-continent intestinal replacement of the bladder. Although each procedure has specific indications, advantages, and disadvantages, all aim to improve the quality of life in patients undergoing cystectomy (1). The Kock, Mainz, and Indiana pouches are the most common heterotopic continent reservoirs; these are mainly created in young patients with associated urethral stenosis or urethral malignancies. Compared with the orthotopic neobladder, the heterotopic continent reservoir offers a lower incidence of urinary incontinence and better day- and night-time continence (2). This report describes a novel heterotopic continental ileal reservoir in which the ileum is used as a urinary bladder; the appendix is incorporated into the ileal wall as an efferent tract, based on the Mitrofanoff principle. This innovative approach offers a potential treatment option for selected cases of bladder cancer.

## Case presentation

The novel heterotopic ileal reservoir was developed at our hospital between December 2021 and June 2022, and was applied in 3 patients (1 woman and 2 men) who had undergone radical cystectomy and standard lymphadenectomy for bladder cancer. The surgical procedures were performed after obtaining informed consent from the patients. A life expectancy of >5 years was achieved in all 3 cases. All patients had good cardiorespiratory and cognitive function, no intestinal lesions, and good renal function, which helped to maintain electrolyte balance and optimal waste excretion). The appendix measured ≥6 cm in length and was wide enough to allow passage of the 14 F spreader and organ-confined tumors without any difficulty. All three cases initially presented with clinical manifestations of gross painless hematuria.

Patient A (female, aged 61 years) was diagnosed with a bladder neoplasm and underwent a transurethral resection in March 2022; histopathology of the resection specimen demonstrated the presence of high-grade invasive urothelial carcinoma. Accordingly, she underwent radical cystectomy with standard lymphadenectomy, followed by the creation of the novel heterotopic ileal reservoir in June 2022. The preoperative contrast-enhanced computed tomography (CT) urograms suggested that the neoplasm was located in the trigone of the urinary bladder and measured 2.0 × 1.5 cm; there was thickening of the bladder wall, and the thicker portion measured 1.48 cm. Therefore, the potential for recurrence was considered and contrast-enhanced CT was performed; The findings showed linear enhancement and a misty perivesical adipose space with multiple fibro-stripe shadows and preoperative right-sided hydronephrosis. Cystoscopy indicated the presence of distinct mucosal hyperemia of the bladder and solid neoplasms on the bladder neck. These findings suggested the preoperative clinical stage was T3N0M0.

Patient B (male, aged 67 years) was diagnosed with a bladder neoplasm and underwent transurethral resection twice between September 2021 and June 2022; histopathology of the resection specimen suggested the presence of low grade papillary urothelial carcinoma. He underwent radical cystectomy and standard lymphadenectomy, followed by the creation of the new heterotopic ileal reservoir in June 2022. Preoperative CT urography showed no remarkable findings and cystoscopy showed the presence of papillary neoplasms of various sizes in the bladder wall and prostatic urethra. Positron emission tomography-CT (PET-CT) indicated a space-occupying lesion on the posterior bladder wall which measured 3.5 × 2.0 cm; the preoperative clinical stage was determined to be T1G2N0M0 (high risk).

Patient C (male, aged 58 years) underwent transurethral resection of the bladder neoplasm four times between March 2019 and January 2022; histopathology of the resection specimens demonstrated a high-grade invasive urothelial carcinoma. He underwent radical cystectomy and standard lymphadenectomy with creation of the novel heterotopic ileal reservoir in January 2022. Preoperative cystoscopy indicated the presence of piecemeal bleeding over an area of 4.7 × 2.4 cm on the left lateral bladder wall with the disappearance of mucosal vascular markings; the texture was crisp and susceptible to bleeding. PET-CT indicated a distinct thickening of the left lateral bladder wall with a neoplasm that had an increased anabolic rate; the factor of neoplasm recurrence was considered. Based on these findings, the preoperative clinical stage was determined to be T2N0M0.

Preoperative radiographic images showed no evidence of distant metastasis, pelvic lymph node enlargement, or hydronephrosis in any of the three patients. Patients A and C received neoadjuvant chemotherapy; however, this could not be administered in both cases due to economic considerations. Patient A received adjuvant chemotherapy and immunotherapy soon after surgery, while patient C refused adjuvant chemotherapy.

### Surgical procedure

**Figure 1:**
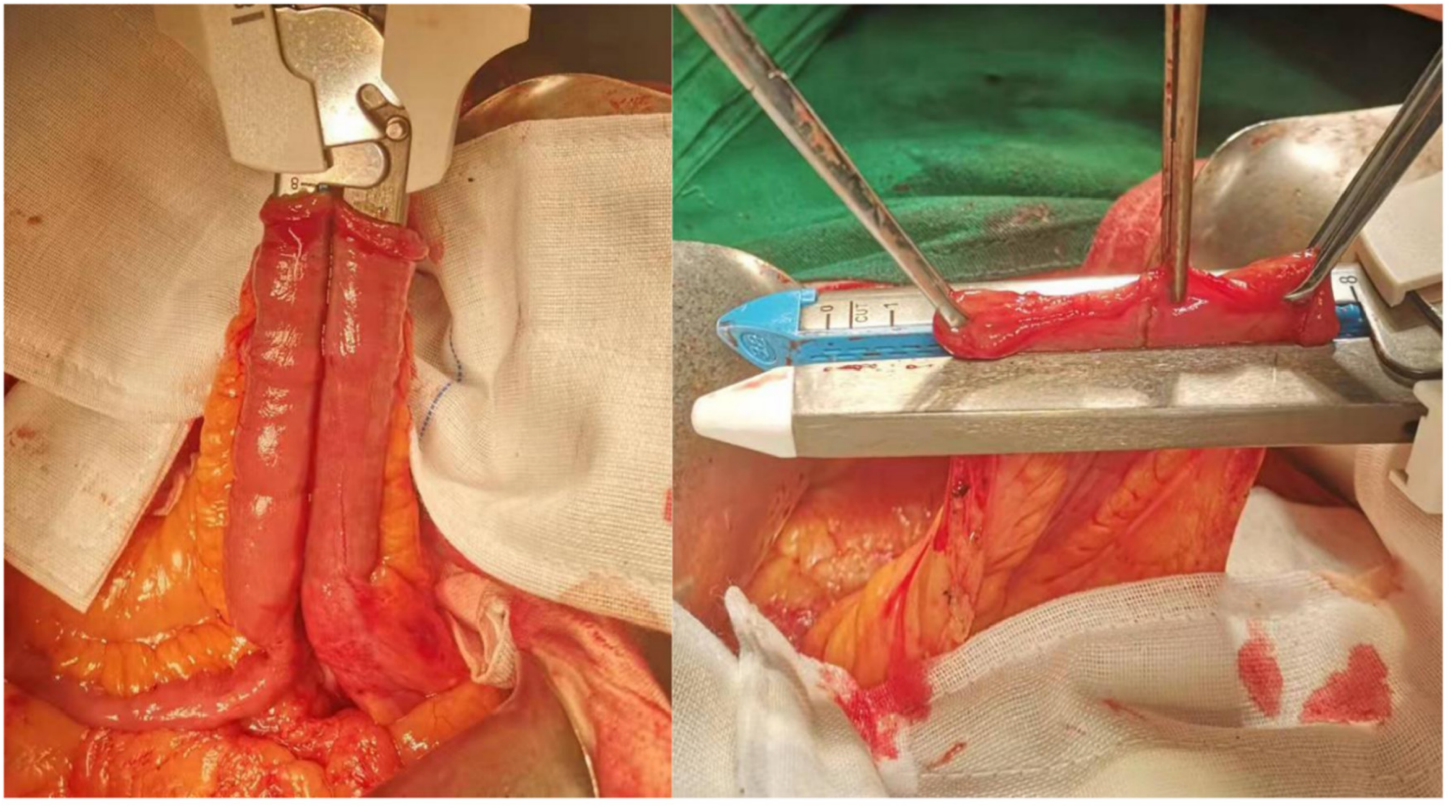
Reestablishing bowel continuity (of the seromuscular layer) using interrupted 4-0 polydioxanone sutures.

**Figure 2:**
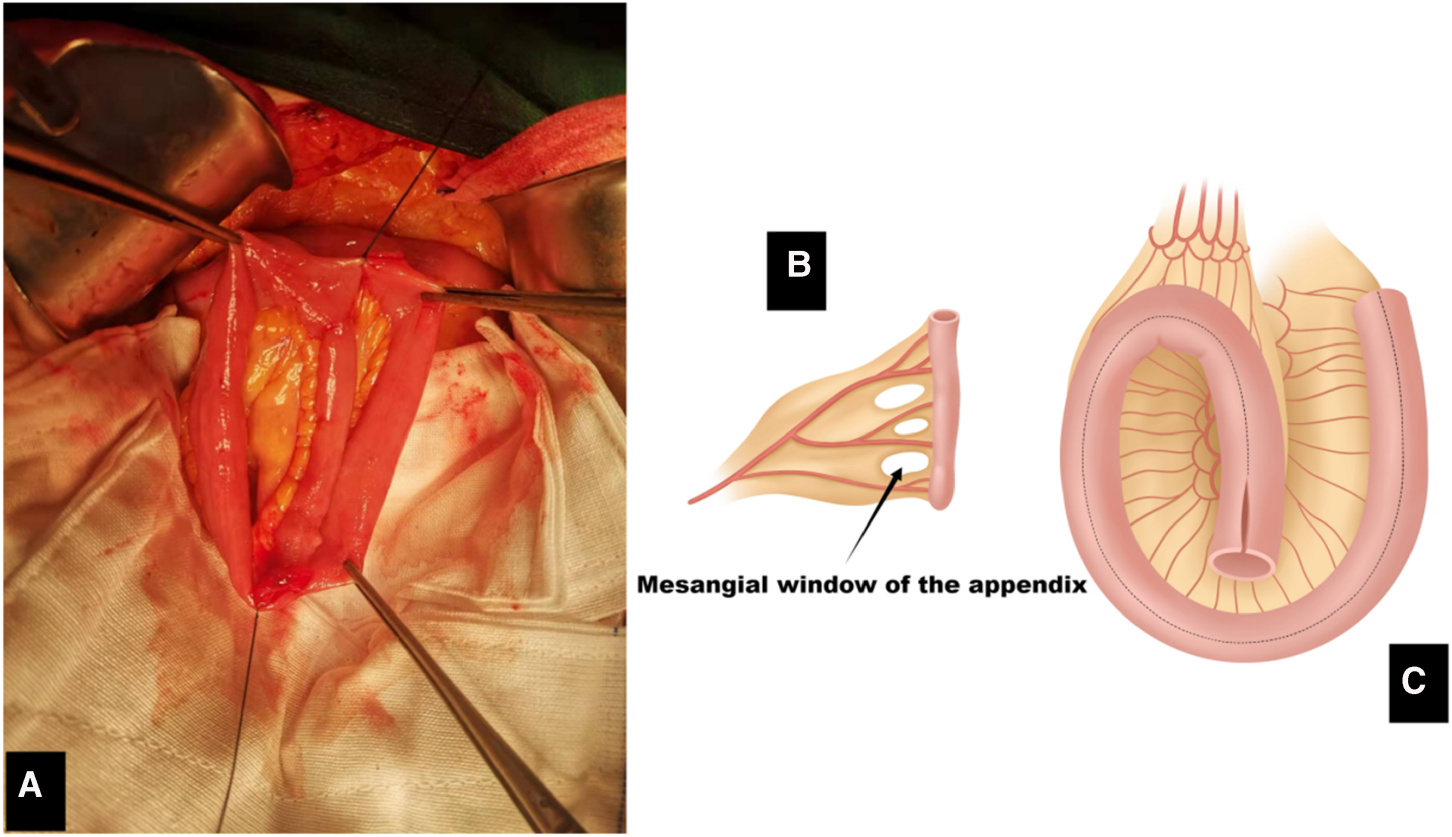
(**A**) Intraoperative image showing the placement of plicating sutures on the ileal pouch. (**B**) Appendix and windows of the mesoappendix. (**C**) The intercepted intestine is formed by counterclockwise rotation as shown in the figure. Suturing was performed from coronal incision for ileal pouch.

**Figure 3:**
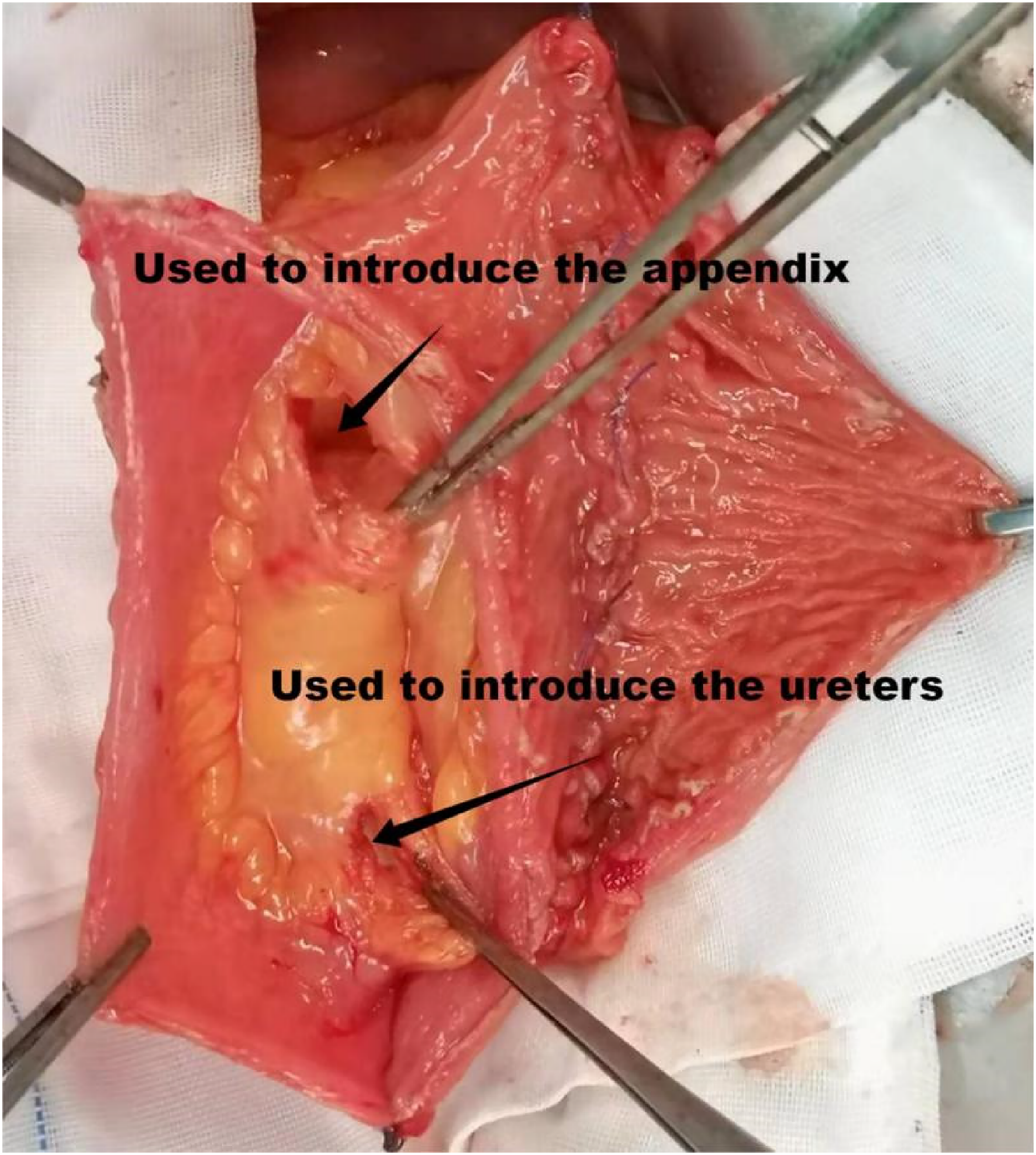
Two windows are opened in the right mesentery of the pouch; the upper and lower windows have been used for passage of the appendix and ureter, respectively.

Open surgery was performed under general anesthesia. The patient was placed in the supine position with the buttocks elevated and a circumumbilical abdominal incision (measuring 20 cm) was performed. Standard lymph node dissection (extending to the bifurcation of the abdominal aorta) after excision of the urinary bladder. In cases with ≤stage T2 tumors, the peritoneum overlying the bladder was routinely preserved for pelvic re-peritonealization. The length of the appendix was confirmed to ensure that it was of the appropriate size and exceeded 6 cm. A 40 cm segment of the ileum was intercepted 15 cm from the ileocecal branch and a stapler was used to reestablish bowel continuity (of the seromuscular layer) using interrupted 4-0 polydioxanone sutures (Figure 1). Several stitches were placed for reinforcement and the mesentery was closed. When the appendix was intercepted, the mesoappendix was protected to prevent any disruption of the blood supply and 3–4 standby windows were opened based on the blood supply of the appendix (Figure 2B). After irrigation of the intercepted intestinal tract, it was coiled to form a shape reminiscent of a continuous circle (Figures 2A,C). The coiled shape was fixed to form 3 segments measuring approximately 13 cm each. The ileum was incised from the anti-mesenteric border and the two adjacent left intestinal walls were sutured with absorbable 2-0 continuous locking mattress sutures and interrupted sutures, with 4–6 stitches placed for reinforcement. The left ureter was crossed over the retroperitoneal space above the inferior mesenteric artery and diverted to the right. The windows were opened in the lower right mesentery (Figure 3); bilateral ureters were passed through these into the space between the two adjacent intestinal walls on the right of the pouch, taking particular care to prevent torsion of the ureters during this procedure. Then, both ureters were anastomosed into the lower portion of the two open adjacent intestinal walls on the right by extramural tunnelling.

### Technique of anastomosis of the ureter and pouch

**Figure 4:**
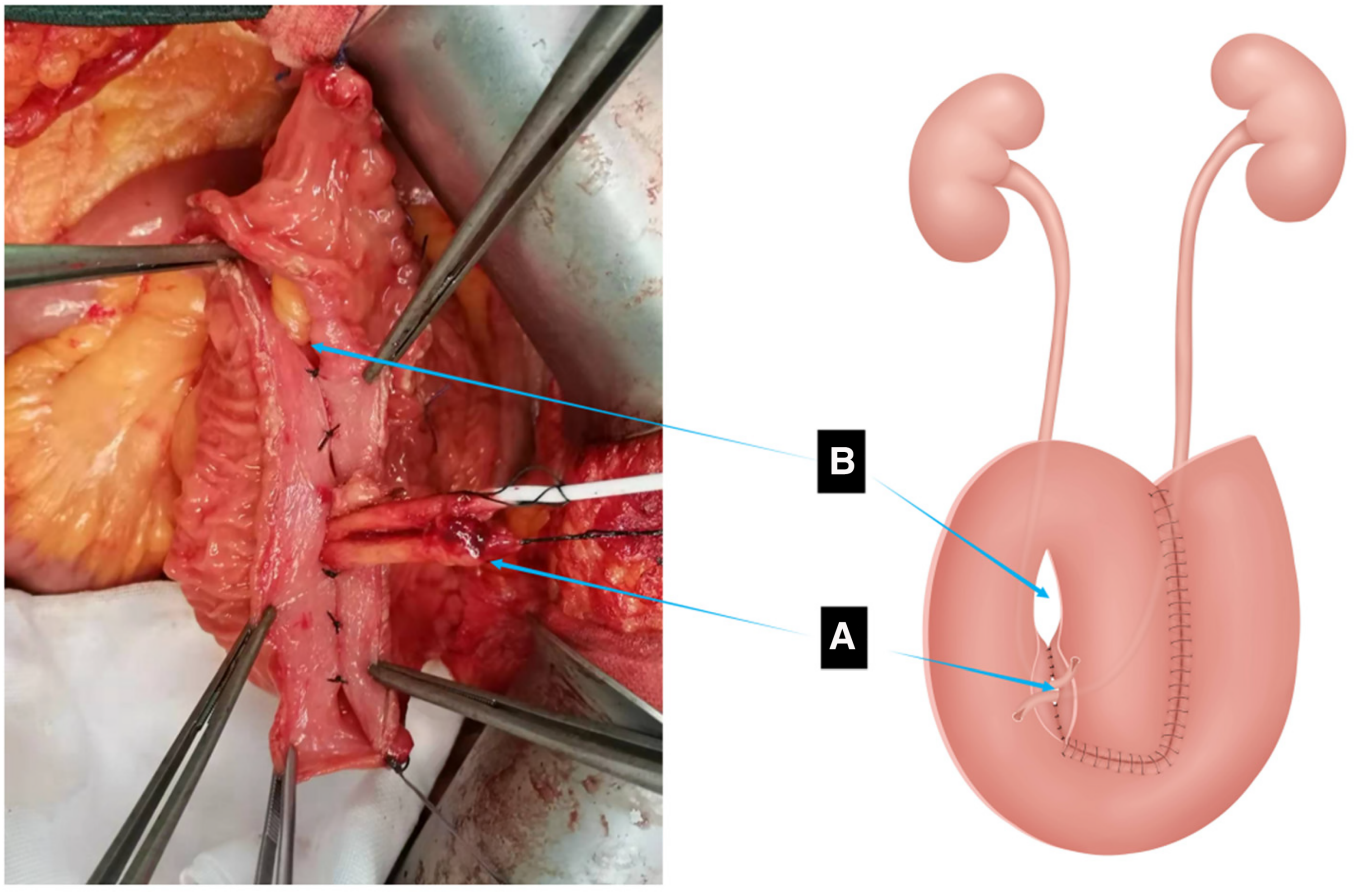
Suturing of the left portion of the pouch is complete. (**A**) Ureter drawn out from the right lower portion of the pouch. (**B**) Site for passage of the appendix.

**Figure 5:**
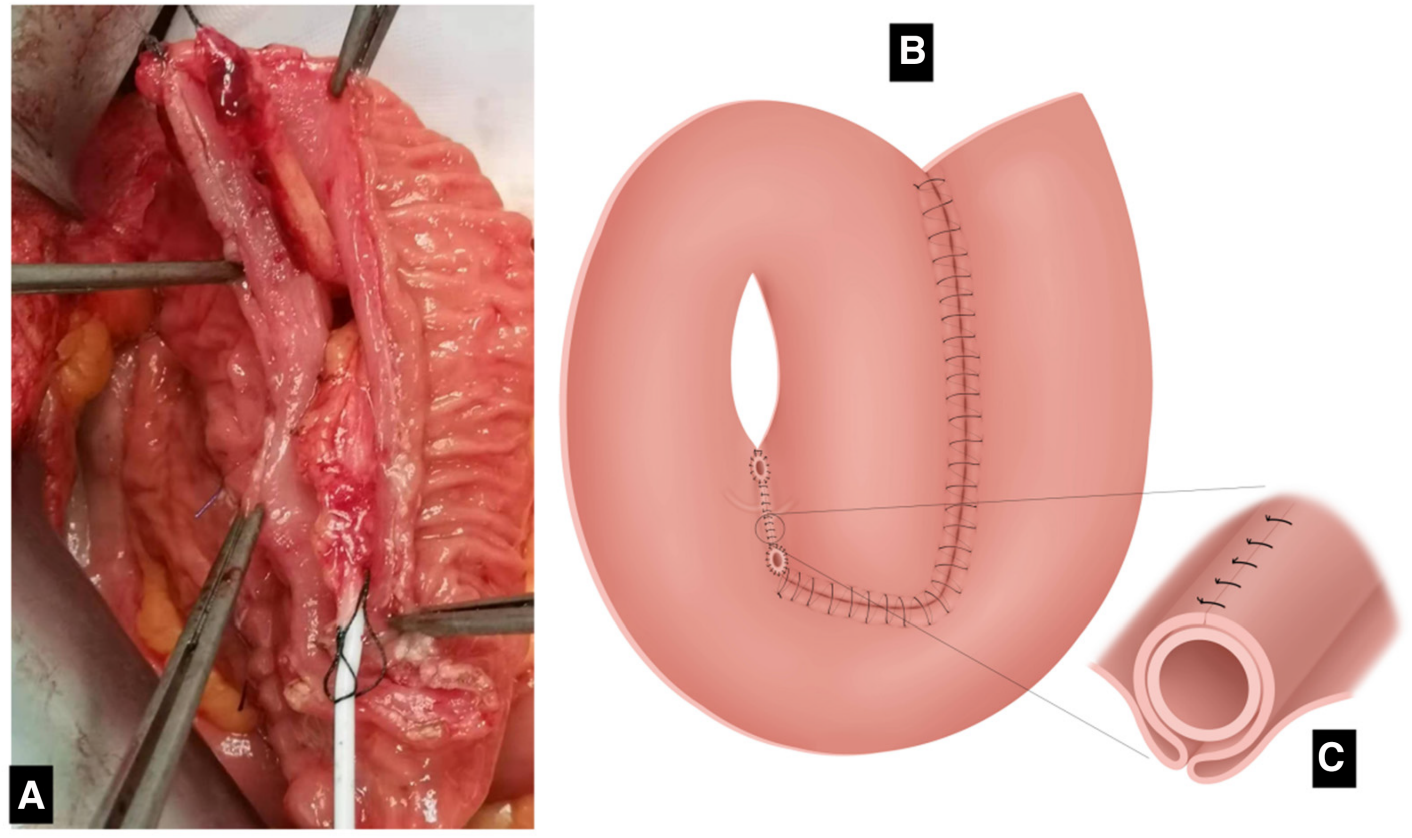
(**A**) Bilateral ureters embedded in the tunnel in cephalad and caudal directions. (**B**) Completion of the anastomosis of both ureters and the right lower portion of the pouch. (**C**) Anastomosis of the ureters and extramural tunnel of the pouch. Interrupted sutures are placed at 5–8 mm from the edge of the ileum opposite the two adjacent segments of the intestinal wall to form the posterior wall of the extra-mural tunnel into which the ureter is buried. Interrupted sutures cover the two segments of intestinal wall above the ureter form the anterior wall of the extra-mural tunnel.

The ureters were drawn approximately 2-cm from between the two adjacent walls to embed into the tunnel (Figure 4). The two adjacent lateral intestinal walls were sutured with interrupted sutures; 4–5 stitches were placed for fixation at a distance of 5–8 mm from the ileal margin to form an extramural tunnel of approximately 2 cm in length. The adjacent sutures were placed at an interval of 5 mm and the left and right ureters were embedded into the tunnel to follow the caudal and cephalad routes, respectively (Figure 5A). A 5 mm incision was placed in the terminal ureter to suture it with the ileal wall using interrupted sutures. The ileal wall overlying the ureters was sutured with interrupted sutures to construct the anterior wall of the tunnel (Figure 5C); particular care was taken to ensure an appropriate distance between the suture and the edge to prevent compression of the ureter during movement. Single J-stents were inserted into both ureters and fixed to their mucosa using 5-0 absorbable suture material. The remaining right lower intestinal wall of the pouch was closed with interrupted sutures (Figure 5B). A 3–4 cm incision was placed in the avascular area of the ileal mesentery on the upper right side near the appendix, which in turn passed through the incision and anastomosed to the upper right part of the pouch by extramural tunnelling.

### Anastomosis of the appendix and pouch

**Figure 6:**
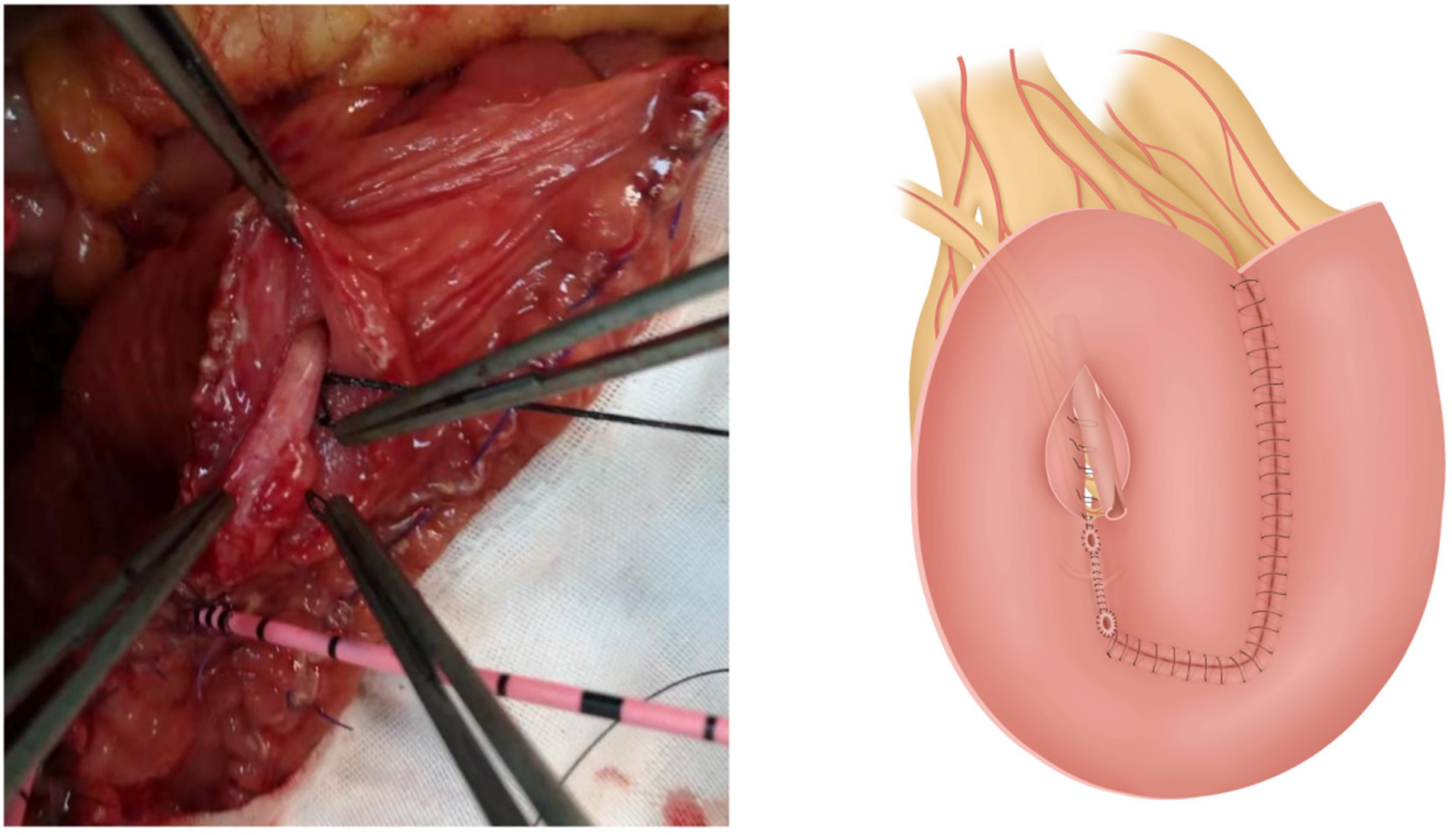
Appendix inserted into the upper portion of the space between two adjacent intestinal walls on the right of the pouch.

**Figure 7:**
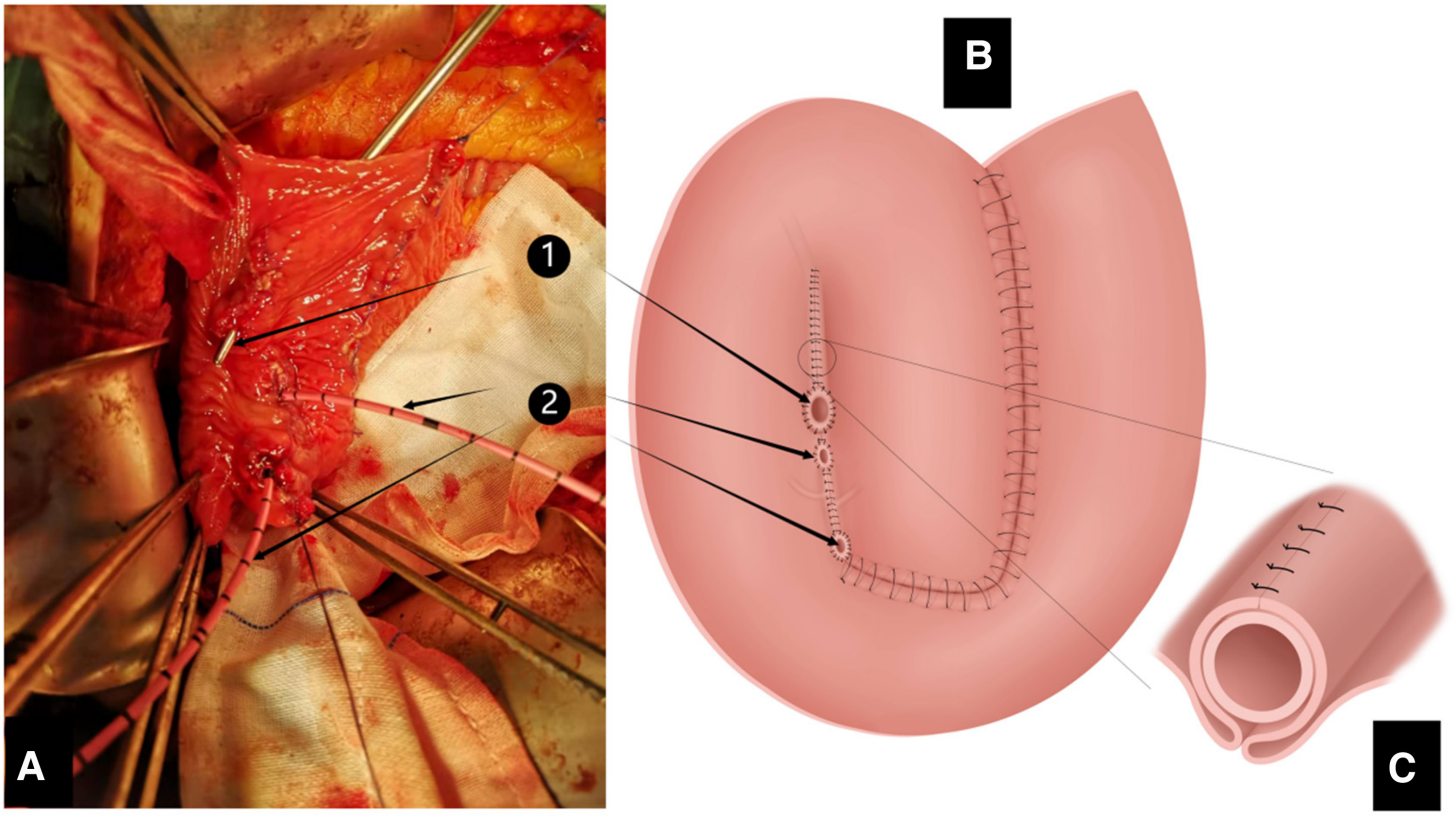
(**A**) Intraoperative image after completion of anastomosis of the appendix and bilateral ureters with the pouch, showing the openings of the appendix and bilateral ureters. (**B**) Completed anastomosis between the appendix and bilateral ureters and the pouch, showing the openings of the appendix and bilateral ureters (1) orifice of appendix; (2) orifices of bilateral ureters). (**C**) Methods of anastomosis of the appendix and extramural tunnel of the pouch (same as in Figure 5C).

**Figure 8:**
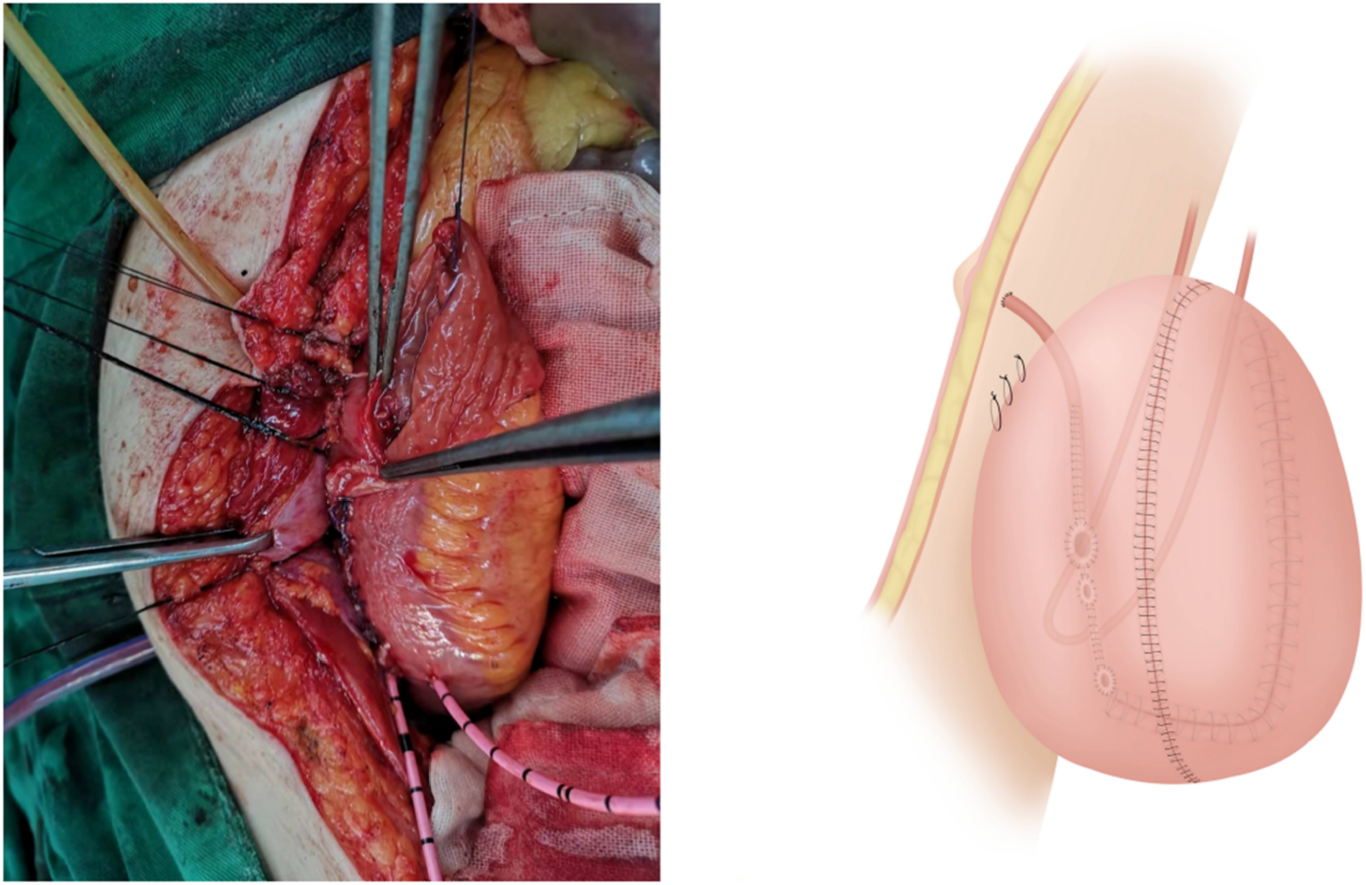
The appendix is anastomosed with the umbilical stoma and the pouch is fixed to the closely adherent right abdominal wall using sutures.

**Figure 9:**
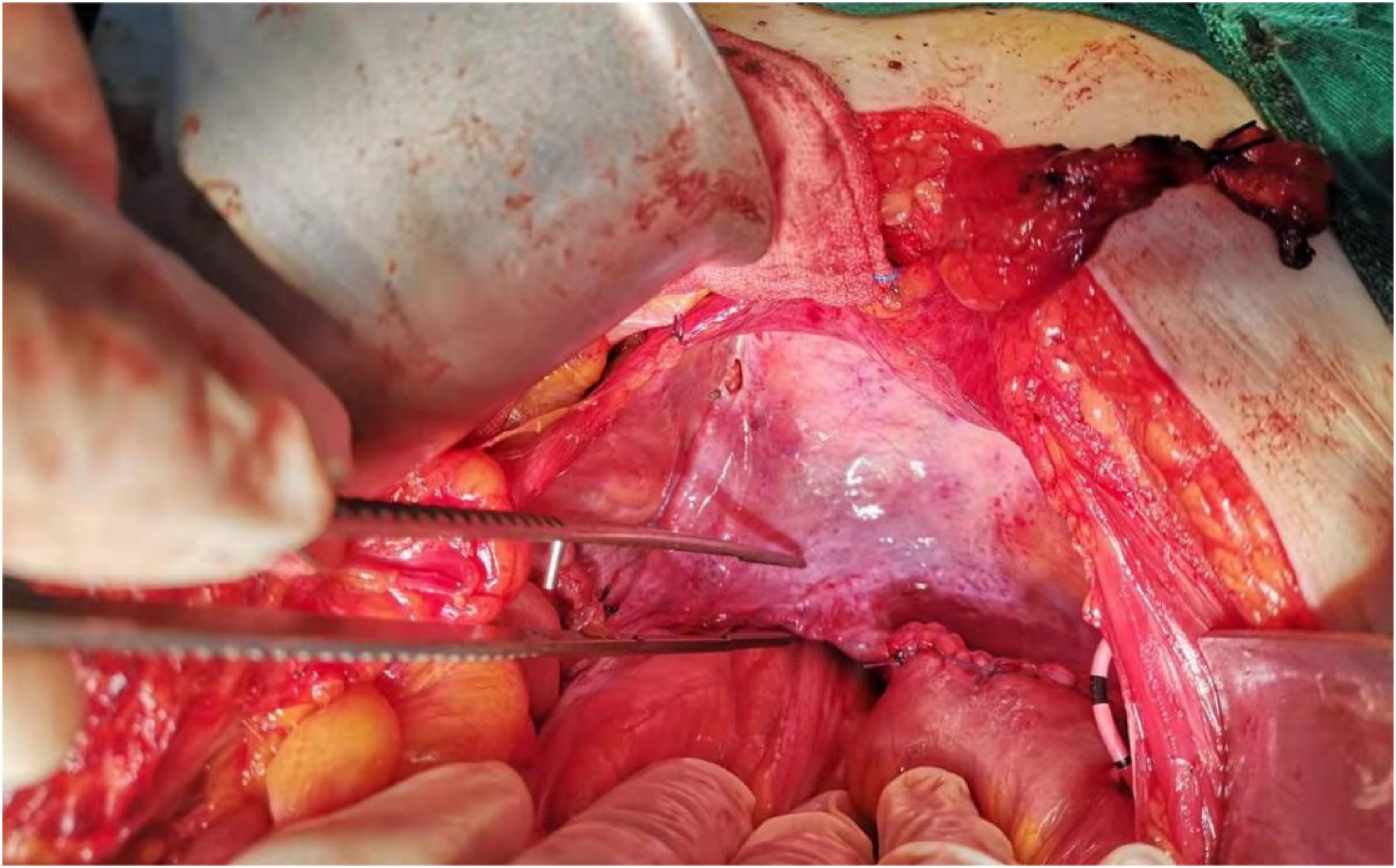
Peritonealization of the pelvic diaphragm has been completed.

The appendix was embedded in the incision of the ileal wall, taking particular care to prevent torsion of the mesoappendix and excessive traction of the blood vessels. The two adjacent intestinal walls were then sutured 8–10 mm from the margin of the ileal wall, the suture lines passing through the windows of the mesoappendix. The appendix was then inserted into the tunnel formed by the above procedures (Figure 6). This not only enabled suturing of the two adjacent intestinal walls together, but also fixed the appendix without disrupting the appendicular blood vessels or causing ischemia of the appendix. The number of stitches depended on the condition of the windows of the mesoappendix; each window was sutured with a single stitch to construct the posterior wall of the tunnel. A 5 mm incision was placed in the terminal appendix, which was dilated using a 14 or 16 F dilator. After dilation, the incision in the appendix was anastomosed to the ileal wall using interrupted sutures. The intestinal wall was then sutured with interrupted sutures to embed the appendix (Figure 7C). The remaining right intestinal wall of the pouch was closed using interrupted sutures (Figures 7A,B); a 14 or 16 F urethral catheter was then repeatedly inserted into the appendix to ensure patency of the lumen. The anterior walls of the pouch were closed from the caudal side using absorbable 2-0 suture material; continuous locking mattress sutures were used and an opening of 2–3 cm was preserved on the cephalad side. Double J-stents were drawn from the anterior wall of the pouch and fixed. A stoma was created in the area of the right abdominal wall closest to the pouch. A 14 F urethral catheter was inserted into the pouch through the stoma to serve as a fistulization tube for the bladder. The lateral peritoneum was then closed from the right paracolic sulcus to the ureter and a transumbilical stoma was created and the appendix was anastomosed and fixed to the umbilical stoma. The periappendiceal ileal and abdominal walls were fixed to reduce the tension exerted on the appendix and the pouch was fixed to the abdominal wall again using 2–3 stitches (Figure 8). In cases of excessive tension on the anastomotic stoma, the ileocecal branch was freed fully to enable fixation to the anterior abdominal wall, thereby reducing the tension; the remaining openings were then closed. In cases where the length of the mesoappendix was too short for allowing connection to the umbilical stoma, a stoma was created in the area of abdominal wall near to the pouch to enable anastomosis. A 14 F urethral catheter was then inserted into the pouch sequentially through the umbilical stoma and appendix and the balloon was inflated as appropriate. Water was injected into the pouch through the fistulization tube for the bladder and the umbilical urethral catheter to check for leakage from the pouch and patency of each drainage tube. Single bilateral J-stents were drawn from the neighboring area of the right abdominal wall to allow *in vitro* fixation with sutures. The abdominal cavity and pelvis were irrigated with large amounts of warm physiological saline and the right parietal pelvic peritoneum was sutured to the pelvic cavity below the ureter using continuous sutures; the lateral peritoneum of the left paracolic sulcus was also sutured similarly. The bottom of the pelvic cavity was covered with the peritoneum that was preserved above the bladder and was fixed to the pelvic floor muscles using interrupted sutures to completely cover the wound of the pelvic diaphragm (Figure 9). Two indwelling drainage tubes were then placed in the pelvis and the integrity of the anastomotic stoma was evaluated. The intestines were then correctly positioned after the surgical count. To close the abdomen, the peritoneum and subcutaneous fat were closed with 2-0 absorbable sutures and the anterior sheath was closed with 2-0 polydioxanone sutures.

## Results

**Figure 10:**
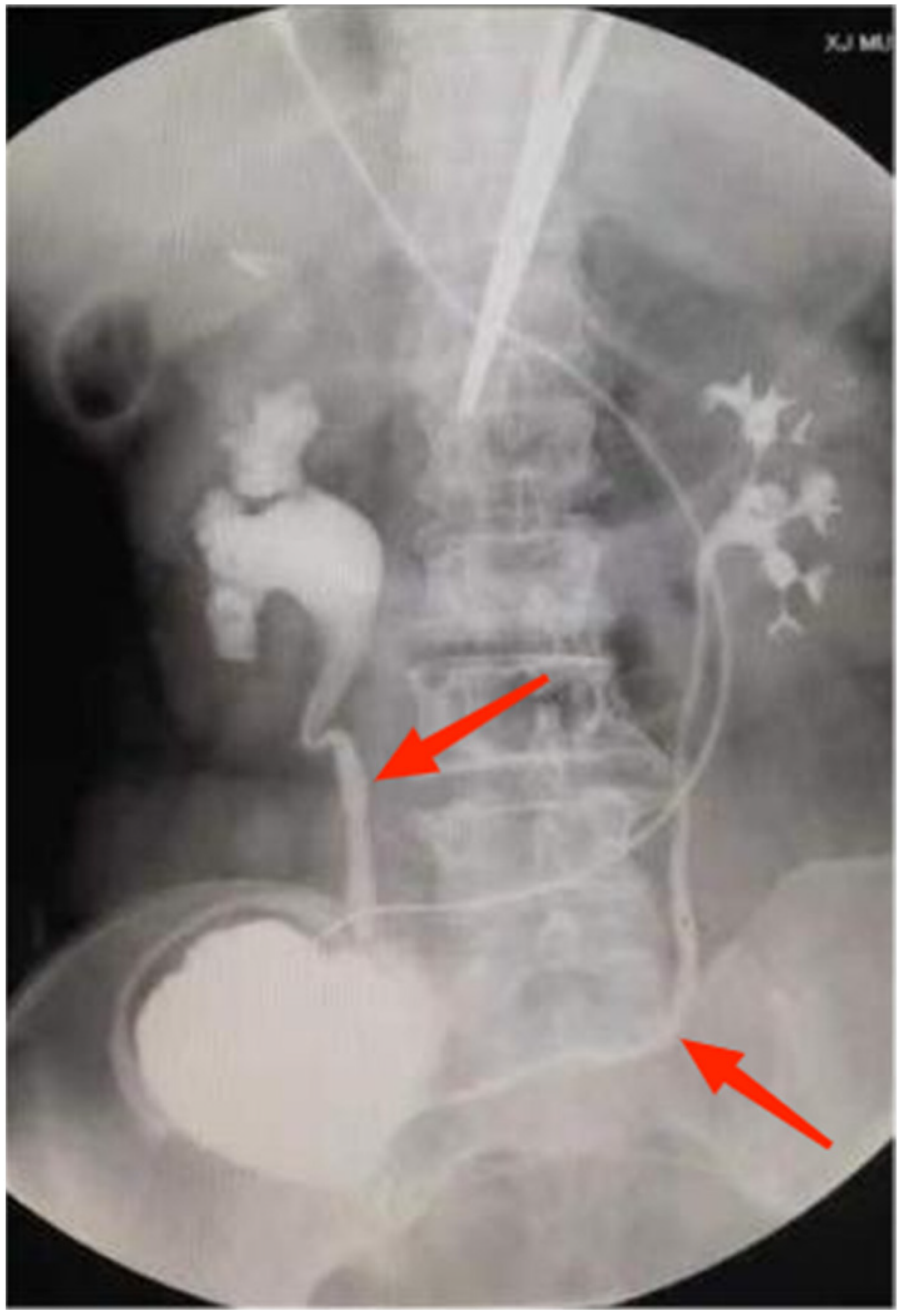
Patient A shows a good bilateral bladder pattern with a volume of approximately 250 ml.

**Figure 11:**
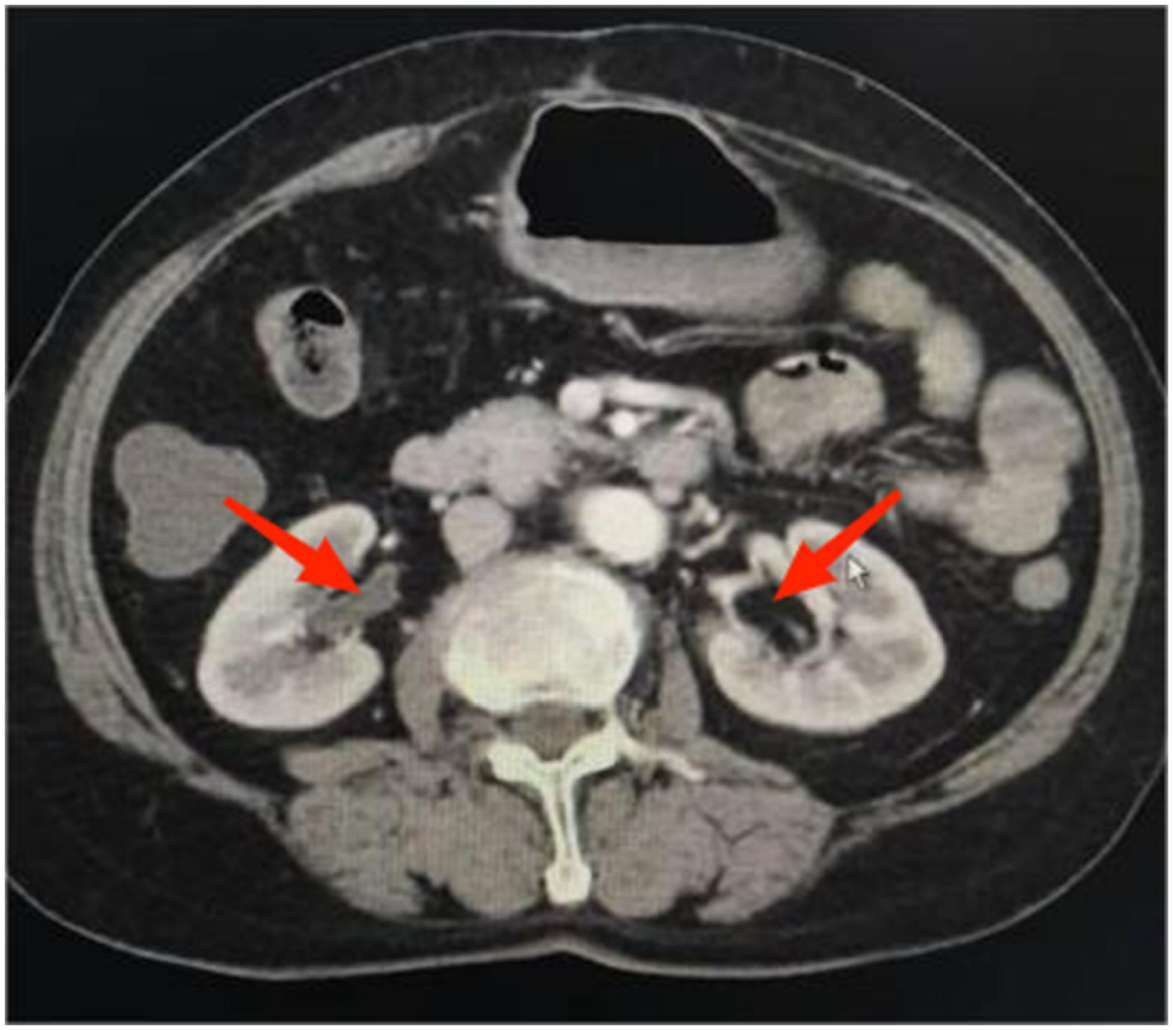
Bilateral ureteral effusion seen in patient A (indicated by the arrows).

Surgery was performed successfully in all patients. The operation time for patients A, B, and C was 410 min, 525 min, and 460 min, respectively. The blood loss for patients A, B, and C was 700 ml, 300 ml, and 500 ml, respectively. The recovery time for intestinal function was 3 days, 5 days, and 5 days for patients A, B, and C, respectively. All patients required postoperative ventilation and the nasogastric tube was clamped for 1 day; In cases with no abdominal distension after oral administration of a small volume of water, the nasogastric tube was removed on postoperative day 2. Oral fluid intake was initiated after removal of the gastric tube. The postoperative hospitalization time was 11 days, 15 days, and 14 days for patients A, B, and C respectively. Subileus occurred in patient B, who recovered after fasting and fluid replacement. Single J-stents were removed at 4 weeks after surgery in all 3 cases and an umbilical urethral catheter was placed at 5 weeks. At-home clean catheterization was performed with a 14 F urethral catheter 4–6 times per day, a regular and fixed amount of water was taken, and bladder irrigation was performed twice weekly. In cases of difficulties with catheterization, a 14–16 F dilator was used for self-dilation. The findings on cystography, which was performed 6 weeks later, were unremarkable and the lumen of the at-home clean catheter was stable; the fistulization tube of the bladder was then removed. At 6 weeks after surgery, 2/3 cases achieved complete continence and umbilical urine leakage occurred in patient A when the catheterization interval exceeded 4 h. At 3 months after surgery, the bladder capacities of patients A, B and C were measured to be 250 ml (Figure 10), 370 ml, and 330 ml, respectively. It should be noted that complete continence was achieved in all three cases (Table 1). Follow-up cystography suggested the presence of bilateral ureteral reflux in patient A, with mild and moderate reflux on the left right sides, respectively (Figures 10, 11); however, this did not require treatment. Patients A, B, and C were followed up for 3, 3, and 9 months, respectively; chest and abdominal CT and cystography performed during this period showed no evidence of hydronephrosis, recurrence, or distant metastasis.

**Table 1:** Perioperative data of the 3 patients.

Name	Sex	Age (years)	Postoperative pathological examination findings	Site with invasion	Neoplasm single or multiple	Postoperative TNM stage	Operative Time (min)
A	Female	61	High-grade invasive urothelial carcinoma	Pulp membrane layer	Single	T3N1M0	410
B	Male	67	Low grade papillary non-invasive urothelial carcinoma	Subepithelial connective tissue	Multiple	T1G2 (high risk group) N0M0	525
C	Male	58	High-grade invasive urothelial carcinoma	Muscular layer	Single	T2N0M0	460

**Table:** 

Blood loss (mL)	Peritonealization of wound	Recovery time of intestinal function	Intestinal obstruction within 3 months	Ureteric Reflux within 3 months	Postoperative bladder volume within 3 months bladder volume (ml)	Continence within 3 months
700	No	After 3 days	No	Bilateral ureteral reflux	250	Complete urinary control
300	Yes	After 5 days	Incomplete intestinal obstruction	No	370	Complete urinary control
500	Yes	After 5 days	No	No	330	Complete urinary control

## Discussion

Ectopic neobladder reconstruction plays a crucial role in the treatment of patients with bladder cancer, as it significantly contributes to prolonging survival and enhancing quality of life (3). Based on experience from our cases and those in the literature, patients need to meet stringent criteria to undergo surgery for novel heterotopic bladder creation. First, they should have good cardiopulmonary health, a life expectancy of >5 years, and good cognitive function (to allow self-management, including regular intubation and catheterization, follow-up, and routine care after surgery). Second, tumors must be limited to the organ without any distant metastases to allow complete resection, as this is an essential determinant of patient prognosis. Third, patients with urethral metastases or positive intraoperative urethral margins who are not good candidates for *in situ* bladder surgery should be considered, as they are particularly suitable for this procedure. Fourth, the length and width of the appendix must be at least 6 cm and 14 F, respectively. A shorter appendix may prevent the new bladder from being appropriately suspended from the abdominal wall. In particular, patients with a thicker layer of parietal abdominal fat require a longer appendix, as an insufficient width may hinder normal catheterization. Fifth, good renal function is necessary, as electrolyte balance and waste excretion must be maintained. In cases of suboptimal renal function, increased pressure in the new bladder may lead to ureteral reflux and further worsen renal function (4). Sixth, the presence of intestinal lesions must be excluded, as this is a prerequisite for the creation of a new bladder using the intestine.

In the past, the ileocolonic or colonic segments were selected as the reservoir for most patients. The colon is relatively fixed and offers a prolonged free time. It also has a considerable number of fat-filled pendants that need to be removed before anastomosis; this prolongs the operation time to some extent and increases the complexity of the operation (5). Furthermore, the colon is rich in bacteria that increase the risk of infection. Colonic pouches increase the risk of hyperchloremic acidosis due to an increase in chlorine resorption and bicarbonate excretion (6). Compared to an ileal pouch, an ileal pouch can save operative time and reduce the incidence of postoperative complications due to a lower bacterial count. Over time, the contractile reactivity of the detubularized ileum becomes more similar to that of the detrusor (7); the ileum becomes more like a reservoir with gradual loss of absorptive function.

The appendix is the most common tubular structure used as the conduit for a heterotopic continent reservoir. Due to the slender lumen and the tension and contractility of the smooth muscle in the appendix wall, which leads to a higher resting pressure relative to that of other conduits, increasing the luminal pressure to three times the pressure of the pouch, it is necessary to insert at least 2 cm of the appendix into the pouch (8). This increases the luminal pressure of the appendix to higher levels than those of the pouch; this is a prerequisite to achieve continence with a heterotopic continent reservoir. It is also necessary for the length and luminal diameter of the appendix to be at least 6 cm and 14 F, respectively (9). Additionally, there should be no significant appendiceal inflammation or stenosis to ensure a reduction in the incidence of stomal stenosis.

In previous classic surgical methods, achieving self-control required incorporating the appendix into the wall of the colon using the Mitrofanoff principle. This principle plays a crucial role in achieving a comprehensive outcome (10). The relatively thin walls of the ileum pose a challenge for the appendix to become embedded within it. In the novel heterotopic ileal reservoir, the appendix is embedded into the ileum through an extramural tunnel to establish a Mitrofanoff mechanism. As there is considerable scope for appendix torsion, this should be avoided by ensuring a secure blood supply for the appendix.

The complications associated with heterotopic continent reservoirs are mainly divided into early and late complications. Shabsigh et al. (11) evaluated 1,142 patients who underwent urinary bladder excision with the creation of a heterotopic continent reservoir and found the incidence of early complications to be 64%; this included gastrointestinal (29%) and infection-related (25%) complications, which are the most common. Compared with other heterotopic reservoirs, the Indiana pouch (in which an ileocecal pouch is used) is associated with a higher incidence of infection-related complications (12). Furthermore, the use of this pouch is an independent risk factor for infections. Late complications mainly include the development of pouch stones, urinary incontinence, stomal stenosis, and ureteric reflux, which has an incidence of 23%–90%. The incidence of ureteric reflux after creation of an Indiana or Mainz pouch, the mainstay of heterotopic continent reservoirs, is approximately 13% (13) and 11.1% (14), respectively. Urinary incontinence, ureteric stenosis, and stomal stenosis are the most common late complications, the latter two being important factors that require surgical revision. Notably, stomal stenosis is the most common complication associated with appendiceal conduits (23.5%) (15). Mild stenosis requires periodic dilatation; in cases of appendicitis due to stomal stenosis or other causes, a urinary catheter needs to be left *in situ* and is removed after recovery subsequent to appropriate anti-inflammatory treatment. In cases where continued use of the appendix is precluded due to inflammation, dilatation failure, or other causes, long-term placement of the cystostomy tube is required. First, peritonealization of the lateral abdominal wall and pelvic cavity prevented intestinal adhesions, accelerated postoperative recovery of intestinal function, and reduced the incidence of intestinal obstruction as much as practicable. Second, during the procedure, the pouch was suspended from an appropriate area of the abdominal wall to ensure adequate blood supply to the ileal mesentery and the appendix; this also prevented the development of appendicitis. Third, we performed ileal extramural tunnelling to implant the ureter into the pouch; this was to prevent ureteric reflux. More follow-up with an evaluation of a larger number of cases will be needed to assess the long-term complications associated with this procedure.

### Limitations

In this study we report a surgical technique for the creation of a novel heterotopic continental ileal reservoir. Although the technique may reduce the risk of developing complications such as infection, ureteral reflux, and urinary incontinence, our procedure has certain limitations. First, a relatively small number of patients were evaluated. This made it difficult to obtain a rapid accurate learning curve and the possible complications associated with this specific surgical procedure. Further studies, including larger sample sizes, are necessary to obtain more comprehensive insights. Second, there are some limitations related to the procedure, which are long, complex, and physically demanding for the surgical team. Third, certain aspects of postoperative management are difficult; these include providing patient instructions for the catheterization technique, catheterization intervals, follow-up intervals, and routine nursing care. Finally, patients were followed for a short period of time; although no long-term complications have been observed, further evaluation will be needed before this surgical procedure can be widely implemented in urology departments.

## Conclusion

In this report, we describe the creation of a novel heterotopic continental ileal reservoir using the ileum as the new bladder and the appendix as the output tract. This approach provides a new treatment option for selected patients with bladder cancer. This procedure should be performed by a trained and experienced urologist, preferably at a professional medical center. This approach may potentially reduce the incidence of complications such as infections, ureteral reflux, and urinary fistulas and is expected to improve quality of life after surgery.

## Data availability

The data that support the findings of this study are available on request from the authors (at wwg0903@163.com).

## Data Availability

The original contributions presented in the study are included in the article/Supplementary Material, further inquiries can be directed to the corresponding author.
